# Enhanced Permeability and Retention Effect as a Ubiquitous and Epoch-Making Phenomenon for the Selective Drug Targeting of Solid Tumors

**DOI:** 10.3390/jpm12121964

**Published:** 2022-11-28

**Authors:** Waliul Islam, Takuro Niidome, Tomohiro Sawa

**Affiliations:** 1 Department of Microbiology, Graduate School of Medical Sciences, Kumamoto University, Kumamoto 860-8556, Japan; 2 Faculty of Advanced Science and Technology, Kumamoto University, Kumamoto 860-8555, Japan; 3 BioDynamics Research Foundation, Kumamoto 862-0954, Japan

**Keywords:** polymer drug, EPR-effect, tumor blood flow, EPR-effect enhancers, BNCT, PDT

## Abstract

In 1979, development of the first polymer drug SMANCS [styrene-co-maleic acid (SMA) copolymer conjugated to neocarzinostatin (NCS)] by Maeda and colleagues was a breakthrough in the cancer field. When SMANCS was administered to mice, drug accumulation in tumors was markedly increased compared with accumulation of the parental drug NCS. This momentous result led to discovery of the enhanced permeability and retention effect (EPR effect) in 1986. Later, the EPR effect became known worldwide, especially in nanomedicine, and is still believed to be a universal mechanism for tumor-selective accumulation of nanomedicines. Some research groups recently characterized the EPR effect as a controversial concept and stated that it has not been fully demonstrated in clinical settings, but this erroneous belief is due to non-standard drug design and use of inappropriate tumor models in investigations. Many research groups recently provided solid evidence of the EPR effect in human cancers (e.g., renal and breast), with significant diversity and heterogeneity in various patients. In this review, we focus on the dynamics of the EPR effect and restoring tumor blood flow by using EPR effect enhancers. We also discuss new applications of EPR-based nanomedicine in boron neutron capture therapy and photodynamic therapy for solid tumors.

## 1. Introduction

The enhanced permeability and retention (EPR) effect is a property of macromolecules larger than 40 kDa or even 800 kDa (even as large as bacteria); these macromolecules include proteins such as albumin and immunoglobulin-G (IgG), polymer conjugates, liposomes, micellar drugs, nanoparticles, and other biocompatible macromolecular compounds [1,2,3,4,5]. The macromolecules tend to accumulate in tumor tissues much more than in normal tissues [1,2,3,4,5]. In 1986, Matsumura and Maeda found that the underlying mechanism of tumor-selective drug targeting is based on the unique characteristics of tumor blood vessels [6]. The causes of the EPR effect are (i) defective tumor blood vessels; (ii) various vascular effectors including nitric oxide (NO), bradykinin, vascular endothelial growth factor (VEGF), carbon monoxide (CO) produced by heme oxygenase-1 (HO-1), and prostaglandins (e.g., prostaglandin E_2_, prostaglandin I_2_) that facilitate extravasation; and (iii) impaired lymphatic clearance, so that macromolecular drugs remain in tumor tissues for extended periods [1,4,7,8,9,10]. The EPR effect occurs not only in primary cancers but also in metastatic cancers including lymphatic, liver, and lung metastases and in inflamed tissues [4,10,11,12]. Different researchers throughout the world have extensively verified the EPR effect in different tumor models as well as in cancer patients [2,13,14,15].

The heterogeneity of tumor tissues is another important issue because the EPR effect depends on tumor blood flow: no blood flow suggests a poor EPR effect or no EPR effect at all [10,11,16,17]. Most experimental tumor models for evaluation of anticancer drugs utilize tumors with small diameters (about 5–7 mm) that are highly vascular and genetically homogeneous, so that the resultant positive outcomes that were expected are observed [10,11,15,16]. In contrast, cancers seen in clinical settings are highly variable—the tumor diameter, for example, can be 2–100 mm or even larger, and clinical tumors can have completely different genetic backgrounds [18,19,20]. Also, advanced human tumors in clinical settings have suppressed blood flow, which often results in the formation of fibrin clots or thrombi and thus unsatisfactory therapeutic effects are seen [21,22,23]. In addition, in tumors with a dysplastic stroma (such as pancreatic cancer), blood vessels may be weakly perfused and even collapsed or obstructed by tumor-associated fibroblasts or pericytes that adhere tightly to vascular walls, which leads to a poor EPR effect and therapeutic failure [10,24,25,26]. These frequent events can mislead researchers to the incorrect view of the EPR effect—that it is not fully observed in human tumors [10,11].

To overcome poor tumor blood flow and enhance delivery of drugs to tumors, our group used NO donors, so-called EPR effect enhancers, which yielded a breakthrough in our investigations [27,28,29]. We found that after administration of various EPR effect enhancers in combination with nanomedicines the delivery of drugs to tumors, as well as the therapeutic efficacy in advanced tumors, increased 2- to 4-fold in various tumor models [10,27,28,29]. These NO donors act as vasodilators to open up tumor blood vessels and increase tumor blood flow, which led to greater drug accumulation in tumors [10,27,28]. Our group also studied another type of EPR effect enhancer—CO donors—which have functions similar to those of to NO donors [30,31,32]. CO is mainly produced in the body during heme degradation, which is catalyzed by HO-1, and has various functions including vasodilation (just like NO) and thus plays a crucial role in enhancing drug delivery to tumors [10,30,32].

This review describes the principle underlying the EPR effect, discovery of this effect, criticisms of the EPR effect, the effect’s heterogeneity, and solutions for different EPR-related issues by using various chemical and physical applications. In addition, we discuss considerations related to radiation therapy, especially photodynamic therapy (PDT) and boron neutron capture therapy (BNCT), which are used for cancer treatment.

## 2. Discovery of the Concept of the EPR Effect

The concept of the EPR effect was first developed during an investigation of the molecular mechanism responsible for the pathogenesis of bacterial infection via activation of protease cascades involving the kallikrein system, which generates bradykinin (kinin) [33,34,35]. Maeda’s group found that less than 1 µg of bacterial protease could induce potent extravasation or enhancement of the vascular permeability of Evans blue-bound albumin in vivo [33,34]. Maeda’s group subsequently found that a similar cascade of kinin generation (e.g., prostaglandin I_2_) were involved in vascular permeability in solid tumors as well as in inflamed tissues [36,37,38]. This finding was extremely significant because at the same time they had been working with the antitumor agent neocarzinostatin (NCS) (12 kDa) to determine how to deliver NCS to cancer tissues as well as metastatic cancers in vivo [3]. 

Basically, they observed the EPR effect during studies of styrene-co-maleic acid (SMA) polymer conjugated with NCS, which they named SMANCS. SMA, a synthetic polymer of 1.2 kDa, has high lipophilicity and was covalently conjugated with NCS via an amide bond [39,40]. Maeda et al. noted that when SMANCS was dissolved in lipid formulations, especially the lipid radiocontrast agent Lipiodol, and injected via a tumor-feeding artery, the tumor/blood ratio of the drug (SMANCS/Lipiodol) at 24 h after injection increased more than 2000-fold compared with the non-modified drug NCS [39,40,41]. Moreover, SMANCS had a longer plasma half-life, about 20 times greater than the parental drug NCS, which was rapidly washed out from blood via bile or urine [40]. At the same time, they validated this unique and important phenomenon by using plasma proteins of different molecular sizes and named it EPR effect [6,42,43]. They found that the nature of extravasation of macromolecules within a specific tumor tissue was very similar to that in inflamed tissue that resulted from bacterial infection or inflammation and that factors affecting the inflammation of infected tissue, such as bradykinin, NO, VEGF, CO, prostaglandins, and many cytokines, were almost the same as those in cancer tissues [3,44]. Matsumura and Maeda first reported this epoch-making phenomenon in 1986 [6].

More than three decades have now passed since the discovery of the EPR effect. This effect has been verified by many researchers throughout the world who utilized various macromolecular drugs including polymer conjugates, liposomes, micellar drugs, and nanoparticles in experimental tumor models as well as in human tumors [10,16,17].

## 3. Criticisms and Misconceptions about the EPR Effect

Certain criticisms have been raised about the validity of the EPR effect [45,46], and some reports indicated that the EPR effect is a controversial concept in tumor drug delivery [46,47,48,49]. For example, these reports stated that nanomedicines did not produce anticancer effects at expected levels and that the EPR effect was not fully observed in clinical settings [46,47,49]. In addition, a recent report offered the opinion that nanomedicines were taken up by the transcytosis pathway and that a very small amount of drugs accumulated in the tumor via the endothelial gap [45]. Transcytosis is an active metabolic process that requires endothelial cells to rearrange their cytoskeleton and form vesicles that help to take up nanomedicines [45]. That is to say, gaps occur infrequently along tumor vessels and then nanoparticles can use active transport through trans-endothelial pathways to enter solid tumors [45]. This opinion was validated by using gold nanoparticles, with particle sizes from 15 to 100 nm, in the Zombie mouse model [45]. To validate this transcytosis-mediated tumor accumulation, however, we believe that other nanomedicines including polymer drugs, liposomes, and stable micellar drugs must be evaluated. Also, a question has arisen about this mechanism: if nanomedicines are taken up via a transcytosis process, why do nanomedicines always show greater accumulation in tumors compared with low-molecular-weight drugs? In addition, to observe the EPR effect or gaps in junctions in tumor blood vessels, permeability factors such as NO, VEGF, and bradykinin must be generated, but in the Zombie model these vascular mediators are barely found.

To address these criticisms and misunderstandings about the EPR effect and nanomedicines, we realized that certain important issues must be clarified. First, during the development of nanomedicines, the stability of the drug during circulation is quite important. For example, after intravenous injection if the active pharmaceutical ingredients, which are covalently linked to polymer conjugates, detach from the polymers or micellar drugs they become unstable in 100% blood, and as a result the nanomedicines behave as low-molecular-weight drugs and no EPR effect is observed [10,11]. Second, certain nanomedicines, especially liposomes (e.g., doxil), are quite stable, so that poor drug release into the tumor resulted and thus a less effective therapeutic outcome was noted [10,50]. Third, many nanomedicines are designed to have positive surface charges to avoid the so-called reticuloendothelial system uptake, but they quickly adhere to vascular walls because their luminal surface is negatively charged; as a result the plasma concentration decreases quite quickly after intravenous infusion [5,10]. Fourth, most tumors in clinical settings are advanced stage and large tumors with embolized blood vessels, so no or very low blood flow is seen [10,28,30]. Also, these tumors are often necrotic and demonstrate no or poor drug delivery [28].

## 4. The EPR Effect Is a Rational and Dynamic Phenomenon for Tumor-Selective Drug Delivery

**Figure 1:**
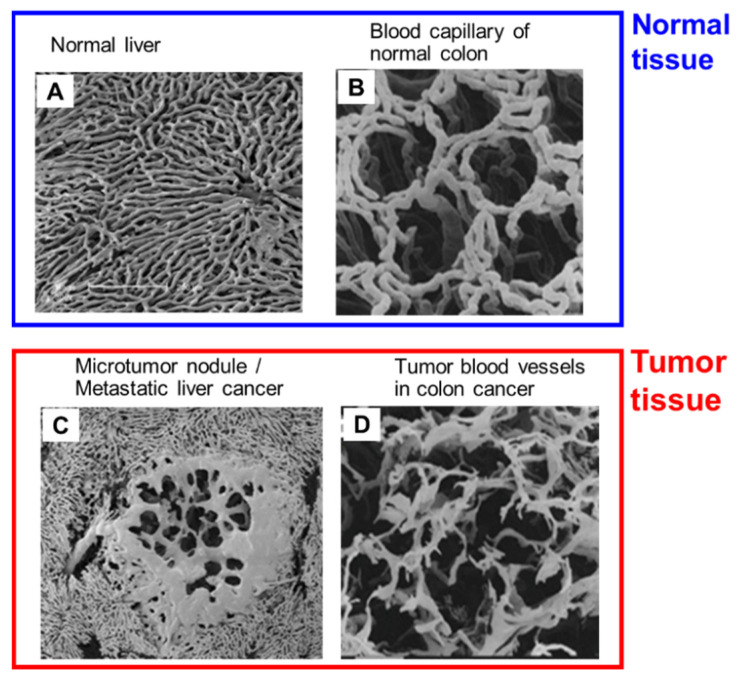
Comparison, via scanning electron microscopy (SEM), of a normal tissue blood vessel (**A**,**B**) and a tumor blood vessel (**C**,**D**) obtained from metastatic tumor nodules in the liver that originated from colon cancer. Blood vessels in normal liver (**A**) and healthy colon (**B**) show clear, smooth tight endothelial gaps and no leakage of polymeric resin. In contrast, tumor vessels, both in liver metastasis (**C**) and in colon cancer (**D**), show leaky blood vessels with irregular features. These SEM images were taken and modified from ref. [44].

Blood vessels in tumors are porous and do not have an architecture with fixed or rigid gaps [4,6,10]. In contrast, normal blood vessels contain tight endothelial cell-cell junctions, and cell-cell junctions can change according to microenvironmental conditions [6,10]. Maeda’s group clearly showed the different architecture in tumor tissue blood vessels and normal healthy tissue blood vessels by means of scanning electron microscopy (SEM) of metastatic tumor nodules in the liver that originated from colon cancer (Figure 1) [44,51]. The blood vessels in normal tissue have clear, smooth tight junctions and no leakage of polymeric resin (Figure 1A,B). In contrast, tumor blood vessels have irregular features, gaps between tight junctions, and polymeric resin leakage at the capillary level or in the early phase of polymeric resin leakage (Figure 1C,D) [44,51]. The gaps junctions of tumor blood vessels will open when blood pressure is elevated or after generation of various vascular mediators, followed by permeability of the tumor substratum [5,44]. Maeda’s group also showed that when SMANCS/Lipiodol was infused into the tumor-feeding artery the drug was delivered selectively to tumors by the virtue of the EPR effect, and this selective delivery was clearly visualized by using computed tomography (CT) [5,29]. In addition, evidence of the EPR effect was acquired by means of radioscintigraphy of tumors with γ-emitting gallium-67 citrate: when this agent was administered intravenously it formed a complex with transferrin (80 kDa) in the plasma that behaved as a nanomedicine. This complex accumulated selectively in solid tumors after 48–72 h and was visualized by using a γ-scintillation camera that provided clear evidence of the EPR effect [4,29].

In addition to Maeda’s group, many research groups throughout the world validated the existence of the EPR effect [15,24,52,53]. Lee et al. recently provided strong evidence for the EPR effect and quantified the effect in human breast tumors including metastatic tumors [13]. They evaluated the EPR effect in 19 patients with HER2-positive metastatic breast cancer by using the ^64^Cu-labeled nanoparticle ^64^Cu-MM-302 (^64^Cu-labeled HER2-targeted PEGylated liposomal doxorubicin) and by imaging via positron emission tomography and CT [13]. They found significant drug accumulation in tumors as a consequence of the EPR effect after 24–48 h of drug treatment in the patients [13]. Ding et al. also analyzed the EPR effect in human renal tumors via X-ray computed tomography and correlated this effect in human tumors with that in animal models [14]. As a surprising result, they found that a considerable EPR effect was present in human renal tumors: more than 87% of human renal tumors showed the EPR effect, with significant diversity and heterogeneity in different patients. All the evidence cited above strongly indicates that the EPR effect is a rational and universal mechanism for tumor-selective accumulation of nanomedicines.

## 5. Heterogeneity of the EPR Effect: An Obstacle to Successful Nanomedicine Therapy in Clinical Settings

**Figure 2:**
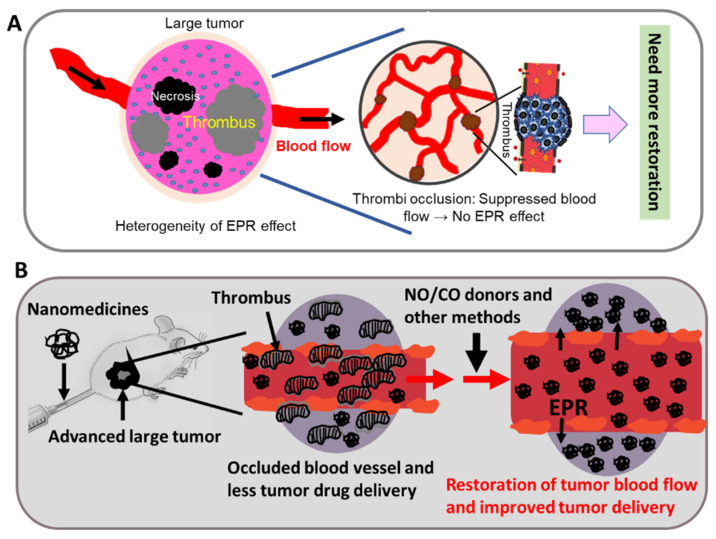
Illustration of an embolized blood vessel in an advanced tumor (**A**) and the strategy used to overcome the suppressed blood flow (**B**). Late-stage tumors possess many necrotic areas, and a fibrin clot resulted in thrombus formation, which blocked tumor blood flow (**A**). In this case no typical enhanced permeability and retention (EPR) effect existed, and additional enhancement of tumor blood flow was needed. (**B**) Mechanism of tumor blood flow restoration by using different methods or EPR effect enhancers.

The heterogeneity of the EPR effect, or embolization of tumor blood vessels, is one issue that mislead researchers about nanomedicines as well as the EPR effect [11,28]. In general, experimental mouse tumors are different from clinical tumors: mouse tumors are smaller (3–10 mm in diameter) and have sufficient tumor blood flow and less or no heterogeneity, so that adequate drug delivery to tumors occurs, based on the EPR effect, and excellent antitumor effects are seen [10,11,28]. In contrast, tumor in clinical settings can be 2–100 mm in diameters or even larger, and these tumors are genetically highly variable or have considerable heterogeneity [11,44]. Also, these late stage tumors have many necrotic areas or occluded blood vessels and no typical physiological blood flow (Figure 2A) [28,44]. We recently determined that the coagulation or thrombogenic system in tumor tissue was highly expressed as tumors grew [37], which resulted in occlusion or embolization of tumor blood vessels and consequently a poor EPR effect, thus reducing the success of cancer chemotherapy in clinic. In this regard, tissue factor (TF) involving the coagulation cascade and chemotactic factors may be involved in obstructing drug delivery based on the EPR effect [10]. Navi et al. recently reported that cancer patients have an increased risk of arterial thromboembolism; however, when this excess risk begins is not clear [21,22]. They studied 374,331 patients 67 years old or older with a new primary diagnosis of breast, lung, prostate, colorectal, bladder, uterine, pancreatic, or gastric cancer or non-Hodgkin lymphoma from 2005 to 2013 and compared the risks of arterial thromboembolic events of cancer groups and no-cancer groups during 30-day periods in the 360 days before the date of cancer diagnosis [21,22]. They found no differences in arterial thromboembolic events from 360 to 151 days before cancer diagnosis between the two populations, but from 150 to 1 day before cancer diagnosis, the risks of arterial thromboembolic events were much higher in cancer patients compared with matched controls [21]. These findings suggest that the nature of tumors in clinical settings is not similar to that of experimental mouse tumors, so additional enhancement of tumor drug delivery is required by restoring tumor blood flow. Figure 2B illustrates the method of tumor blood flow restoration.

## 6. NO Donor-Induced Enhancement of Drug Delivery to Tumors as Well as of Therapeutic Effects

Our group has made great progress in overcoming embolized blood vessels by using NO donors [27,28]. We studied the NO donors nitroglycerin (NG), L-arginine (L-Arg), hydroxyurea (HU), and isosorbide dinitrate (ISDN). Among these agents, NG has been used as a medication for angina pectoris for more than century [54]. NG is administered as an ointment or orally, which selectively generates NO in cardiac infarct tissue and in cancer tissue that is hypoxic and has slightly acidic pH [54,55]. NG first produces nitrite and then converts it to NO by means of nitrite reductase; NO acts as a vasodilator to open up blood vessels [7,56,57].

L-Arg is the substrate of nitric oxide synthase (NOS), especially the inducible form of NOS (iNOS), which is highly expressed in most tumors and inflamed tissues, more than in normal cells [58,59]. iNOS is derived from infiltrated macrophages that produce NO in tumors with relatively high specificity [28,50,58].

HU is used to treat cancer of white blood cells called chronic myeloid leukemia, sickle-cell anemia, cervical cancer, and polycythemia vera. Our group found that HU, like other NO donors, generated NO and increased tumor blood flow [28]. Gladwin et al. and Sato et al. reported [60,61] that, as a possible mechanism, HU generated NO via NOS, because HU is the intermediate in production of NO from L-Arg, and HU may thus demonstrate tumor-selective NO production [10,28,29,60,61].

ISDN is used to treat heart failure and spasms and to treat and prevent chest pain from inadequate blood flow to the heart [62,63]. The molecular mechanism of ISDN is similar to that of other nitrites and organic nitrates: ISDN is converted to NO through an active intermediate compound that activates the enzyme guanylate cyclase [62]. This activation induces the synthesis of cyclic guanosine 3′,5′-monophosphate, which then activates a series of protein kinase-dependent phosphorylation processes in smooth muscle cells, and thus vasodilation occurs [10,29,62,63].

**Figure 3:**
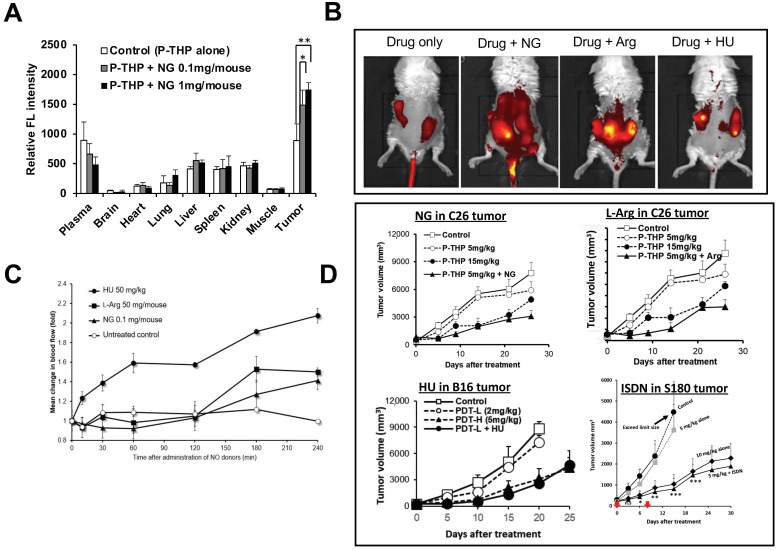
Enhancement of tumor drug accumulation and therapeutic efficacy by using different nitricoxide (NO) domors in implanted tumor models. We first investigated increased drug delivery in various solid tumors that had been generated via subcutaneous administration of cancer cells (2 × 10^7^ cell/mL, 100 μL/mouse) on the dorsal skin. When tumor diameters measured 10–12 mm, the polymer drug was administered intravenously (iv) in combination with NO donors, e.g., nitroglycerin (NG) at the dose of 0.1–1 mg/mouse as an ointment, L-arginine (L-Arg) at 50 mg/mouse intraperitoneally (ip), hydroxyurea (HU) at 50 mg/kg ip, and isosorbide dinitrate (ISDN) at 30 mg/kg, ip. After 24 h of drug treatment, drug accumulation was measured in tumor tissue homogenate by means of fluorescence spectroscopy (**A**) and in vivo imaging by IVIS (IVIS XR; Caliper Life Sciences) (**B**). The improved blood flow in S180 tumors with different NO donors was measured by using a laser Doppler flowmeter (ALF21: Advance Co., Ltd.) (**C**). (**D**) Enhancement of the antitumor effect of various nanomedicines including P-THP [*N*-(2-hydroxypropyl) methacrylamide (HPMA) copolymer conjugated with pirarubicin], PZP [HPMA polymer-conjugated zinc protoporphyrin (ZnPP)], P-PyF (PHPMA polymer-conjugated pyropheophorbide), and SGB-complex [complex formed with styrene-co-maleic acid (SMA) copolymer conjugated with glucosamine and boric acid (BA)] with EPR effect enhancers in different tumor models. Red arrows indicate the (iv) injection point. PDT indicates photodynamic therapy with PZP, L: low dose, H: high dose. Data are expressed as means ± SD. *n* = 5, * *p* < 0.05, ** *p* < 0.01, *** *p* < 0.001. For details, please see Islam et al. [28,29].

**Figure 4:**
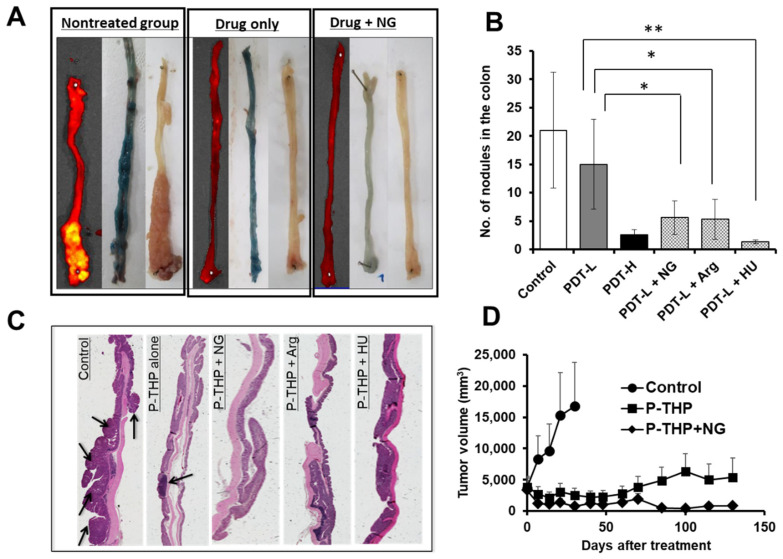
Improvements in therapeutic effects of nanomedicines by using NO donors in chemically induced tumors. Autochthonous colon tumor was developed by administering the chemical azoxymethane (AOM, 10 mg/kg, intraperitoneally) followed by 1 week of drinking 2% dextran sodium sulfate (DSS). At 8–10 weeks after AOM injection, tumor nodules that appeared in the colon were confirmed by examining 2 or 3 randomly killed mice. P-THP at 15 mg/kg iv was infused with NG, and tumor nodules in the colon were visualized by using bovine serum albumin conjugated with rhodamine or Evans blue after 30–40 days of P-THP treatment (**A**). Another nanomedicine, PZP, which was used with light irradiation (PDT) and EPR effect enhancers, produced a similarly improved therapeutic effect (**B**). Enhancement of the anti-cancer effect with NO donors in colon tumors was confirmed by means of macroscopic and microscopic histology (**C**). Arrows indicate tumor nodules in the colon. (**D**) Enhanced therapeutic effect of P-THP with an NO donor in an advanced breast tumor in the rat. Breast tumors were generated by using dimethylbenz[*a*]anthracene (DMBA, 10 mg/mouse, orally), and tumors appeared 12–14 weeks after DMBA administration. Data are expressed as means ± SD. *n* = 5, * *p* < 0.05, ** *p* < 0.01, For details please see Islam et al. [28].

We used combination therapy with different nanomedicines to study the four above-described NO donors in various mouse and rat tumor models. NG was applied as an ointment at the dose of 0.1 mg/mouse [28]. L-Arg at 50 mg/mouse, HU at 50 mg/kg, and ISDN at 30 mg/kg were administered intraperitoneally immediately after drug injection [10,28,29]. To evaluate the enhancement of drug delivery to tumors by these NO donors, i.e., the EPR effect enhancers, we used various tumor models to test five nanomedicines developed by our group: P-THP—*N*-(2-hydroxypropyl)methacrylamide (HPMA) copolymer conjugated with pirarubicin [64]; P-PyF—HPMA polymer-conjugated pyropheophorbide [65]; PZP—HPMA polymer-conjugated zinc protoporphyrin (ZnPP) [66]; SMA-CDDP—the micellar drug cisplatin ion complex with SMA polymer [67]; and SGB-complex—the complex formed with SMA copolymer conjugated with glucosamine and boric acid (BA) [68]. We first investigated the enhancement of drug delivery in mouse sarcoma S180 and colon carcinoma C26 tumor with different EPR effect enhancers and found that tumor drug accumulation increased 2- to 3-fold compared with the nanomedicine alone treatment group, as determined by means of fluorescence spectroscopy and IVIS imaging (the in vivo fluorescence imaging system) (Figure 3A,B) [28,29]. As an interesting result, drug accumulation in normal tissues did not increase significantly after combination treatment with NO donors (Figure 3A) [28]. To support our hypothesis, we measured tumor blood flow with and without various NO donors and found that tumor blood flow increased 2- to 3-fold when combination treatment with NO donors was used (Figure 3C) [28]. We also investigated the therapeutic effects of NO donors in S180, C26, and B16 melanoma tumor models; we found a 2- to 4-fold enhanced antitumor effect with different NO donors (Figure 3D) [28,29]. In addition, we studied the improved therapeutic effects after use of azoxymethane (AOM), which induced autochthonous colon tumors in mice, and dimethylbenzene[*a*]anthracene (DMBA), which induced breast tumors in rats [28]. To produce colon tumors, AOM at 10 mg/kg (0.3 mL/mouse) was injected intraperitoneally into ICR mice, and 1-week later mice were fed 2% dextran sodium sulfate (DSS) in drinking water for 7 days. After 8–10 weeks of AOM administration, mice developed colon tumors [28]. The DMBA-induced breast tumor model was established by administering DMBA: 10 mg of DMBA was dissolved in 1 mL of corn oil and was given orally to SD rats; breast tumors were observed 12–14 weeks after DMBA administration [28]. In both chemically induced tumor models, we also found that combination treatment with nanomedicines and EPR effect enhancers increased the therapeutic effect 2- to 3-fold (Figure 4) [28]. We utilized chemically induced tumor models because these tumors can grow spontaneously and are similar to clinical tumors [10,28].

## 7. Enhancement of the Anticancer Effects of Drugs by Using CO Donors

Our group also developed another method to enhance drug delivery to tumors by using CO donors [30]. CO is a gaseous molecule that is primarily generated in the body during heme degradation, is catalyzed by HO, and has vasodilation functions that are similar to those of NO [30,32]. The inducible form of HO (HO-1) is expressed at high levels in tumors; also called heat shock protein 32, it has antiapoptotic and antioxidant activities and thus facilitates tumor cell growth and survival [10,30,69]. However, detailed mechanisms of vasoregulation induced by CO are not clearly understood.

**Figure 5:**
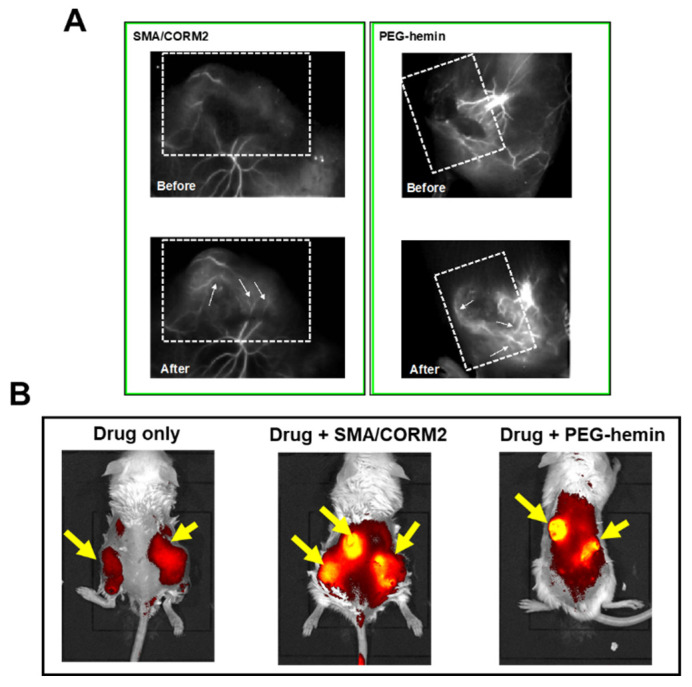
Enhancement of the EPR effect via nano-sized carbon monoxide (CO) donors [SMA/CORM2 and polyethylene glycol (PEG)-hemin] in S180 and C26 tumors. Fluorescence angiography demonstrated improved tumor blood flow after use of SMA/CORM2 and PEG-hemin in S180 tumor-bearing mice (**A**). Increased tumor delivery of P-PyF given at 5 mg/kg iv in the C26 tumor model with CO donors was confirmed by means of in vivo fluorescence imaging (IVIS) at 24 h after drug injection (**B**). Arrows indicate the tumor. Please see Fang et al. [10,30].

Our group developed two nano-sized CO donors: SMA/CORM2 micelles and polyethylene glycol (PEG)-conjugated hemin (PEG-hemin) [31,70]. The reasons for choosing nano-donors are the slow CO release and the tumor-selective accumulation based on the EPR effect [10,32,70]. Of these donors, SMA/CORM2 is an extrinsic supplier of CO and can supply CO slowly because of its nano size [10,30]. The other donor, PEG-hemin, induces HO-1 [31]. Usually, the HO-1-inducing agent hemin is barely soluble in water and has a short plasma half-life and comparatively less accumulation in tumors [30,31]. However, the nano formulation has an improved plasma half-life and drug delivery to tumors [30,31]. Our group showed that both CO donors produced CO more selectively in tumor tissues than in normal tissues, which increased tumor drug delivery 2- to 3-fold by restoring tumor blood flow, as evaluated by fluorescence angiography and fluorescence imaging IVIS (Figure 5) [30]. In addition, when various nanomedicines (e.g., P-PyF) were administered together with these nano-CO donors, the anticancer effect improved 2- to 3-fold in different solid tumor mouse models [30]. These data suggest that CO donors have functions similar to those of NO donors.

## 8. Other EPR Effect Enhancers Used to Improve Drug Delivery to Tumors

To enhance tumor drug delivery, various chemical and physical methods, in addition to NO and CO donors, have been developed [10]. These chemical methods include utilizing tumor necrosis factor α (TNF-α), anti-tissue factor and antibody drug conjugate (anti-TF-ADC), recombinant, micellar forms of tissue plasminogen activator (tPA), anti-VEGF receptor 2 (VEGFR2) antibody, (DC101), Angiotensin II receptor blockers (ARBs), cilengitide, and so on. Physical techniques consist of radiation therapy, sonoporation or ultrasound (US) with microbubbles (MBs), hyperthermia (HT), and PDT. These methods of drug delivery are described below.

TNF-α is a pleiotropic pro-inflammatory cytokine with vascular permeabilizing activity [71]. For example, it is applied during isolated limb perfusion to enhance delivery of chemotherapeutic drugs to tumor tissue [71,72]. Seki et al. showed that TNF-α enhanced endothelial cell permeability and increased drug delivery 2- to 3-fold in the EL4 tumor model and in mice with cerebral brain metastases [71].

Pancreatic cancer is rarely diagnosed at early stages because it often does not cause symptoms until after it has spread to other organs [73]. Despite recent advancements in pancreatic cancer treatment, patients with this cancer have only an 8% chance of 5-year survival [74]. Overexpression of tissue factor (TF) has been seen not only in tumor cells but also in tumor stromal cells, so a cure of pancreatic cancer is not easy [74]. Matsumura’s group reported that anti-TF-ADC, compared with control ADC treatment, significantly enhanced drug accumulation and penetration of tumors in a stromal-rich orthotopic pancreatic cancer model [74,75].

The blood vessels in early-stage tumors are homogeneous, and blood flow is relatively high [22]. In contrast, blood vessels in advanced late-stage tumor tissues in clinical settings are frequently embolized or occluded by fibrin clots, and thus tumor tissues become necrotic, with limited blood flow, and hypoxic [10,68]. The use of thrombolytic agents such as tPA in combination with other nanomedicines or drug carriers may lead to enhanced therapeutic effects by increasing drug delivery close to the solid tumor via fibrin degradation and blood flow restoration [76,77]. Nagasaki’s group reported that administration of tPA together with nanomedicines resulted in 2- to 3-fold-enhanced tumor drug delivery as well as therapeutic efficacy in the A549 tumor xenograft tumor model [76,77].

VEGFR2 is a Kinase insert domain receptor (KDR, a type IV receptor tyrosine kinase). The concept of anti-VEGFR2 was established by developing a monoclonal rat anti-mouse VEGFR2 antibody (DC101) and showing that it potently blocked the binding of VEGF to its receptor, inhibited VEGF-induced signaling, and strongly blocked tumor growth in mice through an anti-angiogenic mechanism [78]. Anti-angiogenic drugs are initially designed for oxygen- and nutrient-deprivation in tumor tissues, however, these agents showed limited therapeutic outcome in clinical setting [79]. Therefore, a strategy to use angiogenesis inhibitors as a tumor blood flow modulator to increase the delivery efficiency of nanoparticles has been developed [80]. In this strategy, an intermediate dose of an anti-VEGF receptor 2 (VEGFR2) antibody, DC101, was applied and successfully normalized tumor vessels to a certain extent such that oriented vascular structure was achieved with increased blood perfusion, decreased vascular density, and reduced necrotic and hypoxic regions [81]. As a possible mechanism Vikash et al. mentioned that, DC101 normalized disorganized tumor vessels by pruning immature vessels and reinforcing the remaining vasculature as well as decreased interstitial fluid pressure (IFP), thus leading to a more uniform and enhanced delivery of a model protein [82]. Coadministration of DC101 with Doxil (~125 nm in size) or Abraxane^®^ (~12 nm in size after dilution in plasma) showed about 3-fold enhancement of tumor drug delivery in breast tumor model [80].

Angiotensin receptor blockers (ARBs), also known as angiotensin II receptor antagonists, are used to treat high blood pressure and heart failure. ARBs can be used to enhance EPR based tumor dug delivery because they amplify the effect of substances like bradykinin, which promote vessel permeability and dilation through the loosening of the fasciae adherens, i.e., the endothelial cadherin mediated intercellular connections [17]. ARBs also modulate the expression of extracellular matrix (ECM) components (e.g., reduction in collagen expression), which leads to vessel decompression and to enhanced EPR effect [83,84]. Various ARBs, for example losartan, which is clinically used to treat chronic kidney diseases and hypertension, but also showed promising preclinical results in cancer treatments, can be used for this purpose [85]. Jain and colleagues showed the combination treatment of losartan significantly improved tumor drug delivery in E0771 and 4 T1 breast carcinoma as well as AK4.4 and Pan-02 pancreatic carcinoma tumor model [85]. A preliminary data indicate that the losartan-based combination therapy led to a decrease in tumor size and in some cases even enabled surgical resection [2]. For example losartan based combination therapy in phase II study showed 2-year overall survival exceeded 60%, and the number of patients where a resection of the tumor was possible after combination therapy exceeded 50%, resulting in 2-year survival in the resected patient population of close to 80% [2].

Wong et al. proposed a strategy named vessel promotion, which focusses on increasing angiogenesis resulting in more vessels and eventually a higher delivery of chemotherapeutic [86]. Cilengitide, a cyclic peptide, which binds to αvβ3 integrins and is usually associated with anti-angiogenesis [87]. This vessel promoting agent was treated in combination with verapamil, a calcium channel blocking agent leading to higher blood flow, resulting in a significant increase of blood vessel perfusion of 10% [2]. The cotreatment of cilengitide, verapamil and gemcitabine, showed a significantly increased mean survival time, approximately doubled compared to gemcitabine only group in a mutagenic mouse model of pancreatic cancer (KPC mice) [2].

Radiation therapy, a quite common cancer treatment, is routinely used in approximately half of all patients with solid tumors [88]. Besides inducing direct antitumor effects, radiotherapy can play an important role in enhancing drug delivery to tumors—both penetration of drugs and their accumulation in tumors [88,89]. Radiation can be used as a physical method to enhance tumor drug delivery: it activated targeted endothelial nanomedicines to induce physical vascular damage related to increased photoelectric interactions [88]. Radiation applied in combination with nanomedicines produced about a 2-fold increase in tumor drug delivery in a human pancreatic tumor model (h-PDAC) and in R3230 mammary adenocarcinomas [88,89].

HT is another physical modality utilized to improve drug delivery to tumors. This treatment relies on local heating of tumors to temperatures up until ~70 °C, and it can be administered in the form of microwaves, radiofrequency radiation, and US [90,91,92]. Mild HT in the range of 39–42 °C promotes perfusion, vasodilation, and vascular permeability, and it enhanced tumor drug delivery, about 2-fold in SK-VO-3 ovarian carcinoma and in the DU145 prostate cancer model [88,92].

Sonoporation can be defined as the permeabilization of cell membranes induced by rapid expansion and compression of MBs after exposure to US [93]. With low-intensity US, MBs disrupted the endothelium and resulted in significantly enhanced tumor drug accumulation in various tumor models [10,88]. That is, when US with MBs was applied with nanomedicines the drug delivery to tumors significantly improved in the highly cellular A431 epidermoid xenografts, the highly stromal BxPC-3 pancreatic carcinoma xenograft model, and clinical pancreatic cancer [10,88,93].

PDT is popular for treating acne and is widely utilized in medicine. We provide details about its application in cancer treatment later in this article. PDT is also applied to enhance tumor drug delivery [94]. Li et al. reported that PDT destroyed tumor-associated fibroblasts and enhanced therapeutic efficacy about 18- to 20-fold in bilateral 4T1, U87MG, MDA-MB-435S, and PC-3 tumor xenograft models [94,95]. 

All the findings described above suggest that enhancement of tumor drug delivery is quite important. Table 1 summarizes currently used EPR effect enhancers.

## 9. Limitation of Using EPR Effect Enhancers

Although, the EPR effect enhancers improve the tumor drug delivery as well as therapeutic efficacy, but there are some limitations of using EPR-enhancing agents. The risk factor can be divided into two categories: systemic adverse reactions based on their pharmacological effects and effects on the tumor due to altering the tumor environment [80]. The major drawback of vasodilators is hypotension [97], and the vasodilator effect as an EPR enhancer should be transient. In addition, NO or CO is a gaseous molecule and they have various bioactivities, both good and bad [10,98]. Under this situation, cautions must be noted because they may cause unexpected side effects when combined with anticancer drugs [80]. NO also plays a role in tumorigenesis, for example, tumor-cell-derived NO promotes tumor progression by induction of tumor-cell invasion, proliferation and the expression of angiogenic factors [99]. The inducible isoform of NOS (iNOS), which generates high concentrations of NO, mediates neoplastic transformation in oncogene- and chemical-induced tumorigenesis models, however conflicting opinion are reported in the literature [98,99].

Angiogenesis inhibitors exhibit some adverse effects on their own such as elevation of blood pressure [100]. However, some reports showed that anti-angiogenic drugs can normalize disorganized tumor vasculature only at the intermediate dose, yet a high-dose can induce the closure of endothelial fenestration and pruned vessels, which leads to a reduction of tumor blood perfusion and thwarts the delivery of anticancer drugs [80,81].

Bharadwaj et al. reported [101] that fibrinolytics agents may increase the chance of cancer metastasis and facilitate the tumor growth. Moreover, systemically administered fibrinolytic agents may cause intracranial hemorrhage, intracranial neoplasm or trauma, hypertension, history of ischemic stroke, and so on [80,102].

Above all results suggest that EPR effect enhancers are involved in several risk, so we need to normalize using enhancers as a combination therapy. Moreover, tumor selective delivery of EPR-enhancer is another important issue need to be clarified, maybe nano formulation of EPR enhancing agent is one possible way for tumor selective accumulation.

## 10. EPR-Based Nanomedicine Breakthrough in BNCT Used in Cancer Treatment

**Figure 6:**
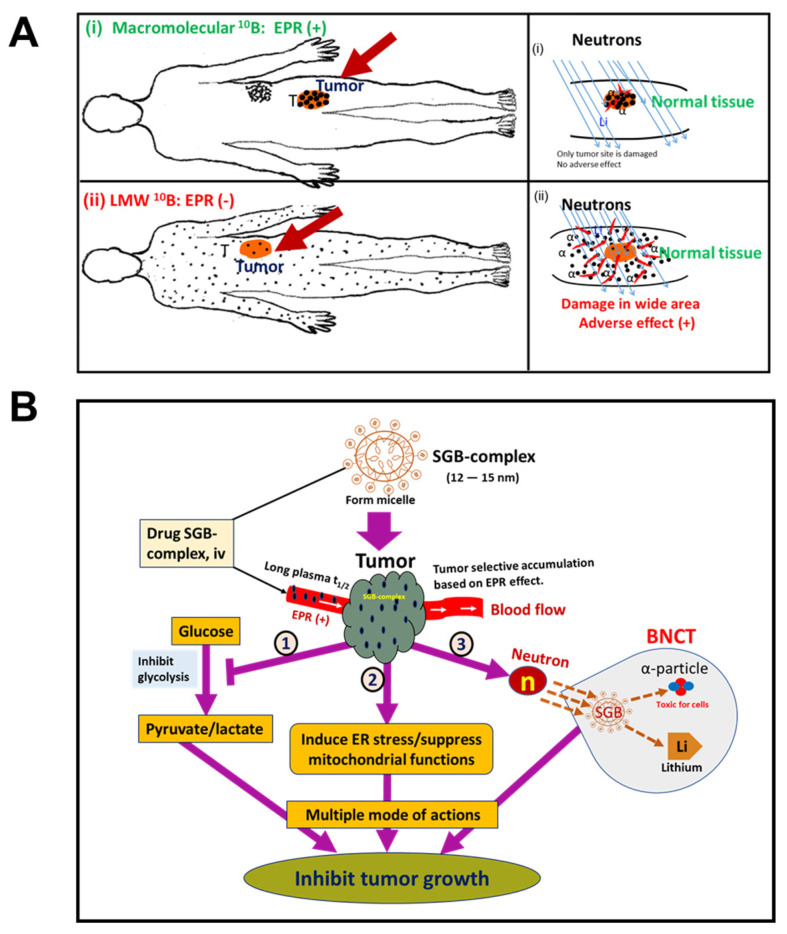
The strategy for successful boron neutron capture therapy (BNCT) (**A**) and multiple modes of actions of the SGB-complex (**B**). The upper panel of (**A**) (i) shows tumor-selective accumulation of a macromolecular borono-drug and α particles only at the tumor site. The lower panel (ii) shows that low-molecular-weight (LMW) drugs are distributed throughout the whole body and produce severe side effects. (**B**) Multiple mechanisms of tumor cell killing by the SGB-complex, which our group developed. ER, endoplasmic reticulum. Please see Islam et al. [70].T he image of (**A**) was taken from the ref. [16] and (**B**) was modified from ref. [68].

BNCT is a cell-selective radiation technique that depends on α-rays emitted from boron-10 (^10^B) atoms when neutrons hit the atoms [103,104]. When boron delivery agents enter tumor tissues and enrich tumor cells, the thermal neutrons trigger fission of boron atoms, which leads to release of ^10^B atoms and then release of α particles (^4^He) and recoil lithium particles (^7^Li) [104,105]. The released α particles are toxic for cells and can result in cell destruction, bouncing out up to 10 µm, which is almost the size of the cells; for this reason this technique is called cell-selective radiation therapy [106]. The first clinical use of sodium borocaptate (BSH) for BNCT was reported by the Japanese scientist Hiroshi Hatanaka in 1960; boronophenylalanine (BPA) was introduced for clinical use by another Japanese scientist Y. Mishima in 1988–1989 [107]. Clinical trials of BNCT for treatment of glioblastoma multiforme and/or melanoma and, more recently, head and neck tumors and liver metastases, with BPA or BSH as the ^10^B carrier, have been performed in many countries including Argentina, Europe, Japan, Taiwan, and the United States [96,103]. Thus, BNCT is not a modern concept, although clinical progress with this method has been quite slow, probably because of the lack of tumor-selective drug accumulation and terrible adverse effects [16,103,108]. Conventional borono-drugs, which are commonly used in clinical settings, are low-molecular-weight drugs that are distributed indiscriminately throughout the body, particularly in the skin, when given intravenously [16,68]. As a result, when neutrons are used to irradiate the whole body these low-molecular-weight drugs produce adverse effects such as skin damage and mucositis, among others [96,103]. Macromolecular drugs, however, have the advantage of tumor-selective accumulation because of the EPR effect [6]. Figure 6A illustrates the problems with conventional BNCT and strategies for successful BNCT. Our group had a breakthrough in our studies to address the clinical drawbacks related to BNCT: we developed a novel multifunctional polymer conjugate drug—the SGB-complex [68]. This SGB-complex formed spontaneous micelle, manifested a single peak by gel permeation chromatography, and had a diameter of 10–15 nm by transmission electron microscopy and dynamic light scattering [68]. We found that intravenously injected SGB-complex bound with albumin during circulation and had a plasma half-life of 8 h in mice; it accumulated in tumor tissues about 10 times more than in normal tissues [68,109]. We developed the SGB-complex primarily for BNCT, but surprisingly we found that it can inhibit cancer cell growth effectively under mildly hypoxic conditions (pO_2_, 6–8%), which resemble tumor microenvironments [48,68]. In addition, the SGB-complex significantly suppressed tumor growth in various mouse tumor models (e.g., mouse sarcoma S180 and colon carcinoma C26) even without neutron irradiation [68]. We hypothesized that, as a possible mechanism, the SGB-complex inhibited glycolysis in cancer cells and affected mitochondrial functions [68,108]. We noted that the SGB-complex released free BA in tumor tissue (pH 5.5–6.5); liberated BA may compete with phosphate in the phosphorylation of glucose to glucose 1-phosphate and may thus inhibit glycolysis in cancer cells [29,68,110]. According to the Warburg effect, under hypoxic conditions cancer cells depend predominantly on energy production via glycolysis instead of the tricarboxylic acid cycle [111]. Thus, suppression of glycolysis in cancer cells will lead to cell death. To confirm our hypothesis, we measured glucose uptake, lactic acid production in hypoxia-adapted HeLa cells, and tumor tissue pH in vivo and found that the SGB-complex significantly inhibited glucose uptake and lactic acid secretion in HeLa cells [68]. Moreover, tumor pH after intravenous injection of the SGB-complex shifted from slightly acidic to neutral, which indicates inhibition of lactic acid production [68]. All the data presented above provide consistent evidence that the SGB-complex inhibited glycolysis in cancer cells. Our data suggest that the SGB-complex is more sensitive in hypoxic conditions than normoxic condition, which means that this nanomedicine is ideal for advanced late-stage cancers, which have low pO_2_, in clinical settings.

We also confirmed excellent anticancer effects of the SGB-complex after neutron irradiation in vitro and in vivo. We used human oral squamous carcinoma cells in vitro and we found, surprisingly, that the cells treated with the SGB-complex at 8 μg/mL (BA equivalent) demonstrated about 16-fold greater cytotoxicity after 10 Gy neutron irradiation when compared with the group treated with the same dose of neutron irradiation alone (no drug) [68]. We also investigated the antitumor effect of the SGB-complex after neutron bombardment in C3H mice bearing human oral squamous cells carcinoma (SCC VII), and we found that the SGB-complex at 10 mg/kg significantly suppressed tumor growth at days 14 and 21 after a single neutron irradiation dose compared with irradiation alone (6 × 10^8^ n/cm^2^/s for 30 min) or compared with the SGB-complex alone treatment group [68,108]. One hallmark result we observed that neutron irradiation of SGB-complex-treated mice did not affect the skin of the mice, nor were other common toxic effects of BNCT treatment (e.g., mucositis, systemic toxicity) [16,68,108]. These common phenomena were seen in treatment with BPA + neutron irradiation or other conventional borono-drugs [16,29,68,103,108]. These results indicate the promising future of the SGB-complex for BNCT in clinical settings. Figure 6B illustrates the multiple modes of action of the SGB-complex.

## 11. The Significant Role of EPR-Based Nanomedicine in PDT

PDT has been known for more than 100 years, but its practical impact in cancer treatment has been negligible [11,112]. PDT is a treatment that utilizes a photosensitizer (PS) followed by light irradiation. When a PS is irradiated by absorbing light at certain wavelengths, the energy level of the PS increases, a photoactivation reaction occurs that causes fluorescence emission and formation of singlet oxygen (^1^O_2_) from molecular oxygen (O_2_), and the energy level of the PS will fall to the ground state [11,113]. ^1^O_2_ is a reactive oxygen that plays a role as an oxidative reagent in cells and can lead to cell killing or mutation by damaging nucleic acids, proteins, and lipids [11,112,113]. Commonly used PSs in clinical settings are Photofrin^®^, temoporfin (Foscan^®^), motexafin lutetium, palladium-bacteriopheophorbide, tri ethyl etiopurpurin (Purlytin^®^), verteporfin (Visudyne^®^), talaporfin (Laserphyrin^®^), and some modified versions of PSs [114]. All are low-molecular-weight compounds, which do not manifest the EPR effect, and thus they are distributed throughout the entire body, especially the skin [11,65]. When light is irradiated, ^1^O_2_ generation occurs wherever the excitation light is accessible in the presence of a PS, and thus a hypersensitivity reaction of the skin is observed [10,11]. However, nano-PSs can solve this problem, because of the tumor-selective accumulation of drug by virtue of the EPR effect [10,11,66].

**Figure 7:**
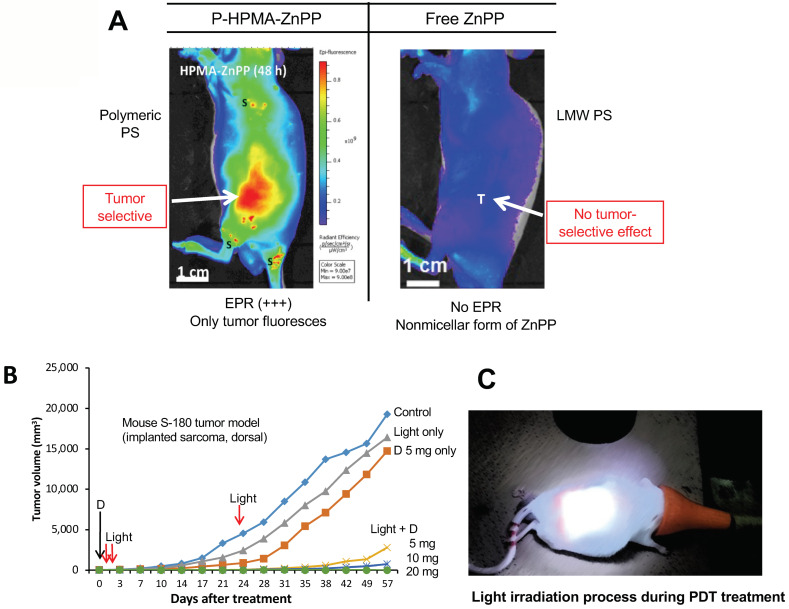
Advantages of EPR-based nano-sized photosensitizers (PSs) for PDT. When a macromolecular PS, PZP, was injected iv, intense drug accumulation occurred in the tumor after 24 h, as shown by IVIS fluorescence imaging (**A**, **left image**). In contrast, the LMW drug free ZnPP did not show any significant tumor drug accumulation (**A**, **right image**). (**B**) An excellent antitumor effect of PZP was seen in the mouse sarcoma S180 tumor model, especially with endoscopic light irradiation. “D” indicates the drug with iv PZP treatment; red arrows indicate light irradiation. (**C**) Light irradiation process with mouse tumor. Please see refs. [10,11,66].

Maeda’s group developed several nano-PSs including PZP [66], P-PyF [65], PEG-ZnPP) [115], SMA-ZnPP [116], and others. They found that free ZnPP did not produce any significant tumor accumulation at 24 h after intravenous injection, but nano or micellar forms of PSs showed markedly higher drug accumulation in various mouse and rat tumors, as shown by fluorescence imaging IVIS [65,66] (Figure 7A). In addition, these forms produced excellent antitumor effects in different xenograft mouse tumor models (e.g., mouse sarcoma S180, colon carcinoma C26, B16 melanoma) (Figure 7B) and AOM/DSS-induced autochthonous colon tumor in mice (Figure 4B) [10,27,28,66]. All the above findings suggest that nano-PSs have a potential for use in successful PDT for cancer by minimizing adverse effects.

## 12. Concluding Remarks

In this review, we described the rational and dynamic EPR effect, which was discovered by Professor Maeda and Dr. Matsumura in 1986 [6]. Some criticisms and arguments about the EPR-based tumor-selective accumulation of nanomedicines have appeared in the literature [45,46,49], but we and many other research groups throughout the world have addressed such misconceptions with considerable pre-clinical and clinical data [10,13,14,117,118,119,120]. One critical issue involves embolized or occluded tumor blood vessels, which lead to mistaken interpretations of the EPR effect [10]. To observe EPR-mediated tumor drug delivery, additional enhancement of tumor blood flow is necessary, because the EPR effect depends mainly on tumor blood flow [1,10]. We have discussed the various methods used to improve tumor blood flow, which Table 1 summarizes.

In addition, we have discussed the problems and solutions related to two important techniques in cancer treatment: BNCT and PDT. The advancement of these treatments in clinical settings is negligible because of the lack of active pharmaceutical ingredient entry into tumor tissue [11,65]. Nano formulations of borono-drugs or nano-PSs may be solutions for a successful strategy that utilizes BNCT and PDT for cancer treatment.

## Figures and Tables

**Table 1:** EPR effect enhancers used to improve drug accumulation in tumors and their modes of action.

Methods	Drugs/Agents	Tumor Model	Outcome (Augmentation)	Brief Mechanisms
**Vascular mediators**	**NO generating** (i) NG (ii) L-Arg (ii) HU (iv) ISDN (v) Sildenafil	**Xenograft tumor** S180, C26, B16, 4T1 **Chemically induced** AOM/DSS-induced colon tumor and DMBA-induced breast tumor	2- to 5-fold	Open tumor blood vessels as a vasodilator and thus improve drug delivery to tumors [10,27,28,29,30,96]
	**CO generating** (i) SMA/CORM2 (ii) PEG-hemin	S180, C26, B16	2- to 3-fold	Functions similar to those of NO donors [10,30]
	**Others** (i) Tumor necrosis factor-α (TNF-α) (ii) Anti-tissue factor-antibody drug conjugate (anti-TF-ADC) (iii) Tissue plasminogen activator (tPA) (iv) anti-VEGF receptor 2 (v) Angiotensin II receptor blockers	(i) EL4 (ii) Pancreatic cancer (iii) A549 (iv) Breast tumor (v) 4T1, AK4.4, E0771, Pan-02	(i) 2- to 3-fold (ii) Significantly (iii) 2- to 3-fold (iv) 3-fold (v) Significantly	(i) Increase endothelial cell permeability [71] (ii) Enhance penetration capacity [74] (iii) Restore blood flow via fibrinolysis [76] (iv) Normalized disorganized tumor vessels by pruning immature vessels [78,79,80,81,82] (v) promote vessel permeability and dilation through the loosening of the fasciae adherents [2,79,80,81,82,83,84,85]
**Physical methods**	(i) Radiation therapy (ii) Hyperthermia (iii) Ultrasound (US) with microbubbles (MBs) (iv) PDT	(i) h-PDAC, R3230 (ii) SK-VO-3, DU145 (iii) A431, BxPC-3 (iv) 4T1, U87MG, MDA-MB-435S, and PC-3	(i) 2-fold (ii) 2-fold (iii) Significantly (iv) 18- to 20-fold	(i) Induce physical vascular damage related to photoelectric interaction [80,88] (ii) Improve perfusion, vasodilation, and vascular permeability [80,88] (iii) Disrupt endothelium [80,88] (iv) Damage tumor associated fibroblasts [80,88]

## Data Availability

Not applicable.
